# Case of Vaccine

**Published:** 1801-04

**Authors:** T. Pole

**Affiliations:** Leadenhall Street


### *Case of Vaccine, by* Mr. T. Pole, Surgeon.

THE Vaccine Matter I received fometime fince from my friend Dr. Jenner, I employed for the inoculation of Frances Cannon, a girl, about 14 or 15 years of age, of the fanguine temperament; I inferted it into both arms in the ufual place. I examined the punctures on the third day after the infertion, and reported that the matter had taken effect; from there being a flight prominence, an inflammatory rednefs extended a fmall diftance round the puncture. I faw her again on the fixth day, the elevation, or inflammation of the part, had not at all increafed fince the firft examination; I then was ready to conclude it had not taken effect. On the eighth day I faw her for the third time, and from there being then no advancement of the inflammation, and the natural Small-pox being in the houfe where fhe lived, the family were folicitous to have her inoculated from that patient, left fhe fhould take it naturally; to which I confented, and it was accordingly done in one arm only, about an inch and a half from the vaccine puncture. On the third day from the variolous inoculation, I faw the patient, and was fully convinced that each of the two vaccine, and the variolous inoculation, had all taken effect; this circumftance gratified me much, confidering it as one of the faireft opportunities for the vaccine difeafe to fhew its effects upon the variolous; the patient being firft inoculated with the vaccine, and on the eighth day with the variolous, and was alfo expofed to the contagion of the latter. Both the difeafes went on together without interfering with each other, until the inflammation extending from the vaccine puncture furrounded that of the variolous, and the fyftem became evidently affected by febrile fymptoms, attended with head-ache. On the fifth day the variolous infection was arrefted in its progrefs; the puncture was elevating, and the inflammatory circle was fpreading in its ordinary courfe till that time, after which it made no farther progrefs. A very fmall quantity of matter formed in its centre, about three times the dimenfions of a common pin's head, and then died away: the vaccine puftule took its courfe in all refpects, excepting its remaining almoft inactive for the firft eight days, of which I do not remember an inftance occurring before; but if there is any thing in this cafe worthy of more public notice, Dr. Jenner is at liberty to make what ufe of the communication he may think proper; and if he will oblige me with any obfervations on the cafe, I doubt not of their utility to myfelf and others, on fo new and important a difeafe, to which he has fo worthily called the ferious attention of at leaft both Europe and America.

*Leadenhall Street, March* 17, 1801. T. POLE.

